# Hoffa fracture of medial unicondylar and bilateral in a man: a rare case

**DOI:** 10.11604/pamj.2015.20.382.6092

**Published:** 2015-04-17

**Authors:** Samba Koné, Abdoulaye Bana, Stanislas André Touré, Seydou Koné, Ange-Sylvain Allou, Adelaide Natacha Kouassi, Akue Gerard Koffi, Innocent M'bra Kouamé

**Affiliations:** 1 Service de Traumato-Orthopédie CHU de Cocody, Côte d'Ivoire; 2 Institut Nationale d’ Hygiene Publique (INHP Abidjan), Côte d'Ivoire; 3 Service de Traumato-Orthopédie CHU de Bouaké, Côte d'Ivoire

**Keywords:** Hoffa fracture, medial condyle, bilateral topography

## Abstract

Intra articular Coronal fracture of the femoral condyle (Hoffa) is rare-especially that of medial condyle. We report the case of a patient who had a bilateral Hoffa fracture of the medial condyles (type 33 - B3, Orthopaedic Trauma Association) occurred as a result of an accident at work (worker mover). Lesions were treated with the functional and orthopaedic approach. At follow- up of 8-month functional score was excellent with normal flexion, an absence of laxity, an absence of pain and an unlimited market. Through a review of the literature the mechanism, the diagnostic methods and therapeutic will be analysed.

## Introduction

Coronary, isolated from the dorsal portion of the femoral condyle fracture was first described by Friedrich Busch (1844-1916) and not as always assumed by Albert Hoffa in 1904 [1]. Unicondylar knee fractures are rare lesions [1]. Although the often easy diagnosis can be difficult because only poorly visible on a standard x-ray of poor quality or non-displaced fracture. Present here a case report of Hoffa fracture of medial and bilateral unicondylar in a man. A brief discussion on the mechanism lesions and the fundamental principles of the treatment will be exposed.

## Patient and observation

**Figure 1:**
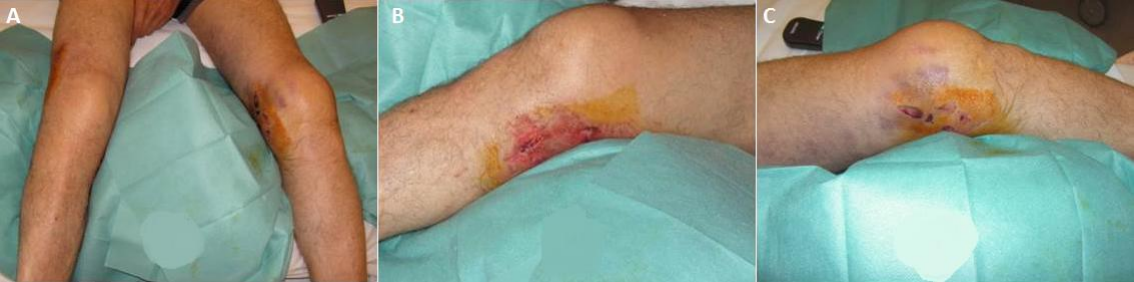
Local appearance of skin lesions of knees; (A) front view; (B) medial face right knee; (C) medial face left knee

**Figure 2:**
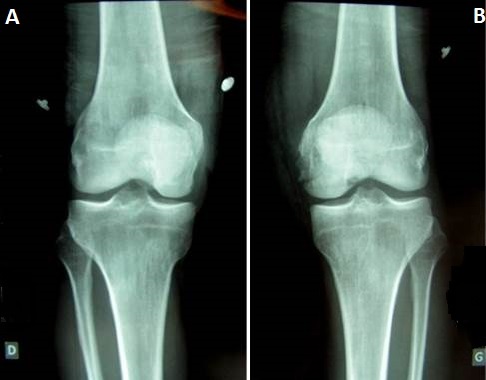
Antero-posterior radiographs of knees joint showing only of the left knee Hoffa fracture of the femoral medial condyle (A) right knee; (B) left knee

**Figure 3:**
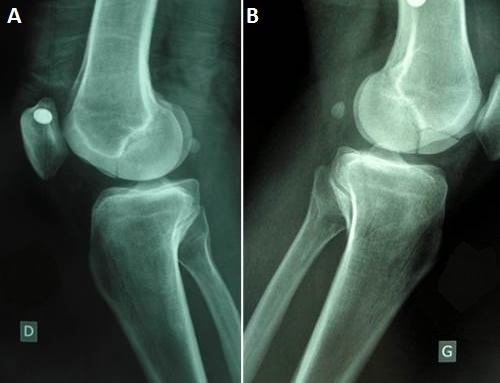
Medio-lateralradiographsof knees joint showing a bilateral Hoffa fracture of the medial femoral condyle and fracture suspicion of left tibia plateau. (A) right knee; (B) left knee

**Figure 4:**
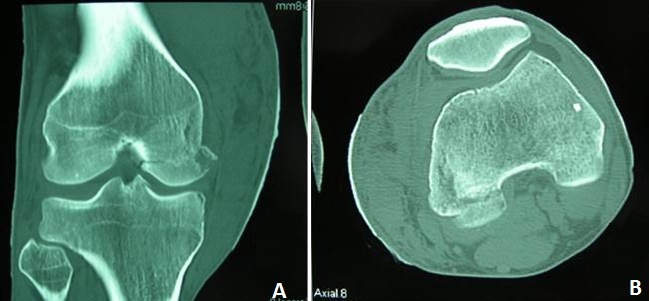
CT scan of right knee had enabled the discovery of a non-displaced fracture of the medial condyle. (A) coronal; (B) axial

**Figure 5:**
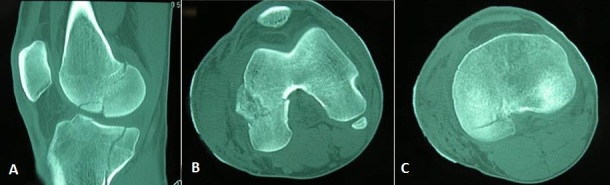
CT scan of left knee and tibia plateau had allowed to objectify the left medial condyle fracture and also the discovery of an associated fracture of left tibia plateau. These were not displaced. (A) Sagittal; (B) condyle axial; (C) axial

It was Mr. B P. Age from 44 years, admitted to the Emergency Department for injuries knees following an accident at work. It is a moving worker who allegedly received while working on his knees, a cab office heavy mode of injury has a direct impact on the knees at 20 degrees of flexion. Review painful swelling of the knees with a patellar shock at the level of the knees (hemarthrosis), more intense pain sitting at the level of the internal compartments of the knee. No there was no vasculonervous, but important lesions of varying degrees of complications (Figure 1). These skin lesions were at the level of medial and lateral sides of the knees. Because of the pain we had sought signs of laxity. The initial AP and lateral x-rays showed a bilateral Coronal fracture of the medial condyle (left and right knee: Figure 2, Figure 3). The scanner showed Coronal fractures type Hoffa of the two medial condyles left and right associated with a fracture of the left tibia plateau (Figure 4, Figure 5).

**Figure 6:**
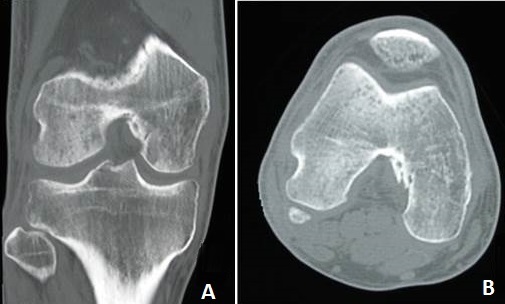
Control CT scan of right knee is highlighting a perfect consolidation of bone lesions. (A) coronal; (B) axial

**Figure 7:**
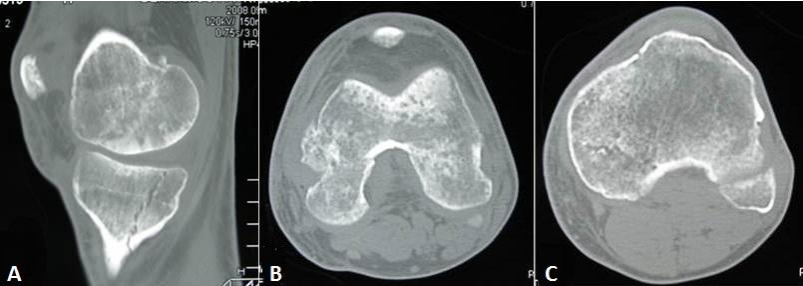
Control CT scan of left knee showing a perfect consolidation of bone lesions. (A) sagittal; (B) axial femoral condyle; (C) axial tibia plateau

According to AO classification [2] the lesion of condyles (Figure 1) was Type 33 -B3 and the lesion of left tibia plateau was type 41-C1. The patient support was primarily a functional method by a strict bed rest; knees were immobilized in knee braces. Locally we had carried out regular dressings and cry therapy. Also a treatment medical was associated with painkillers, of the anti-inflammatory and anticoagulant. Physiotherapy and maintains it muscle was carried out at the patient´s bed. Control scanners were regularly (J8 and J15) made, they did not show secondary displacement. After the healing of skin lesions, we had immobilized knees by the cruro-pedious resins for 04-week. At the end of this period, we proceed with the removal of the resin. The fact of removing is able to thaw the resumption of physical therapy for a period of 6 weeks. Note that the patient kept splints of knees outside the rehabilitation sessions. The market and on the members support him were banned until bony healing. One followed radio-clinic was undertaken externally every month until consolidation. The fuse was acquired in 3 half months. The control scanner is highlighting a perfect consolidation of bone lesions (Figure 6, Figure 7). At final follow- up of 8-month functional assessment of the patient score was excellent with normal flexion, an absence of laxity, an absence of pain and an unlimited market.

## Discussion

Partial articular, coronal plane fractures of the posterior part of femoral condyles are rare and account for less than 1% of distal femoral fractures. Generally results from trauma secondary to motor vehicle accidents or a fall from a height [3]. High velocity injuries such as road traffic accidents (motor vehicle accidents) are the most common mechanism of injury [3, 4]. The peculiarity of our case is the mode of injury and the circumstance of trauma. Mode of injury in this case was a workplace accident (worker mover) and non-traffic trauma. Circumstance is was lower-energy trauma (crushing knees by a mass heavy). The medial condyle is quite special for our patient as the bibliography suggests that the lateral condyle is most often injured [5, 6]. Probably because of following physiologic genu valgus that puts greater compressive stresses on the lateral side. Frontal impact on a flexed knee is more likely to involve the outer aspect resulting in shearing force on the posterior part of lateral femoral condyle.

Bilateral Hoffa fracture [7] or unicondylar medial Hoffa fractures [4] are well described. Medialunicondylar and bilateral Hoffa fractures are extremely rare [8]. It's the second interest and one of specific of our case. The mechanism of injury for our patient can be constrained compression on the medial side of knees associated with a varus knees being flexes. Usually there is no deformity excepted big swollen and hemarthrosis of knee. Standard x-ray of knee can contribute to the diagnosis but this interpretation can be difficult while poor quality snapshot. In practice these injuries can be poorly viewed radiography as in our case on standard radiographs.

In these cases the contribution of the CT scan is undeniable: confirms diagnosis, enjoys travel, detect comminution, and research associated injuries. Associated lesions have been described in literature [4, 9–11] but fracture of tibia plateau (ipsilateral injury to the left knee) associated has not yet been reported. As they are intra-articular fractures, they should be treated by anatomical reduction, rigid internal fixation and early mobilization to restore function. Some authors [3, 7, 9] recommend open reduction to restore normal condylar anatomy and rigid internal fixation. The articular surface is exposed through a medial or a lateral approach, depending on which condyle is involved. Hoffa fractures are typically reduced and fixed with anterior-posterior or posterior-anterior oriented screws [7, 10, 11]. Problems of this surgical approach are infectious hazards, devascularization also fragment necrosis. Nowadays treatment with endoscopic mini invasive (Arthroscopic) surgery may reduce its risk.

Conservative treatment of Hoffa fracture has some risk: secondary displacement, prolonged immobilization give rise to myoatrophy and joint stiffness. In our case we realized an orthopaedic treatment due to septic risk due to skin lesions and the absence of displacement. This treatment is binding but can lead good functional results. The non-bloody treatment must be mainly reserved for displaced and stable fractures.

## Conclusion

In conclusion, we describe a case of a bilateral unicondylar medial Hoffa fracture treated successfully with orthopedic method. Peculiarity of our case is: the scarcity of its localization, unusually, let wasn't result of motor vehicular accidents, it is a closed injury. CT scan allowed diagnosis and associated injuries.
